# MicroRNA Expression and Identification of Putative miRNA Targets in Ovarian Cancer

**DOI:** 10.1371/journal.pone.0002436

**Published:** 2008-06-18

**Authors:** Neetu Dahiya, Cheryl A. Sherman-Baust, Tian-Li Wang, Ben Davidson, Ie-Ming Shih, Yongqing Zhang, William Wood, Kevin G. Becker, Patrice J. Morin

**Affiliations:** 1 Laboratory of Cellular and Molecular Biology, Hopkins Medical Institutions, Baltimore, Maryland, United States of America; 2 Department of Pathology, Johns Hopkins Medical Institutions, Baltimore, Maryland, United States of America; 3 Norwegian Radium Hospital, Oslo, Norway; 4 Research Resource Branch, National Institute on Aging, Baltimore, Maryland, United States of America; Deutsches Krebsforschungszentrum, Germany

## Abstract

**Background:**

MicroRNAs (miRNAs) represent a class of small non-coding RNAs that control gene expression by targeting mRNAs and triggering either translation repression or RNA degradation. Emerging evidence suggests the potential involvement of altered regulation of miRNA in the pathogenesis of cancers, and these genes are thought to function as both tumor suppressors and oncogenes.

**Methodology/Principal Findings:**

Using microRNA microarrays, we identify several miRNAs aberrantly expressed in human ovarian cancer tissues and cell lines. *miR-221* stands out as a highly elevated miRNA in ovarian cancer, while *miR-21* and several members of the *let-7* family are found downregulated. Public databases were used to reveal potential targets for the highly differentially expressed miRNAs. In order to experimentally identify transcripts whose stability may be affected by the differentially expressed miRNAs, we transfected precursor miRNAs into human cancer cell lines and used oligonucleotide microarrays to examine changes in the mRNA levels. Interestingly, there was little overlap between the predicted and the experimental targets or pathways, or between experimental targets/pathways obtained using different cell lines, highlighting the complexity of miRNA target selection.

**Conclusion/Significance:**

Our results identify several differentially expressed miRNAs in ovarian cancer and identify potential target transcripts that may be regulated by these miRNAs. These miRNAs and their targets may have important roles in the initiation and development of ovarian cancer.

## Introduction

MicroRNAs (miRNAs) are 21–23 nucleotide regulatory RNAs processed from 70–100 nucleotide hairpin pre-miRNAs [1], [2]. The miRNA are incorporated into a ribonucleoprotein complex called RNA-induced silencing complex (RISC) and guide the RISC to the target mRNA [3]. Binding of the miRNA to the target mRNA 5'UTR can downregulate gene expression through inhibition of translation or increased RNA degradation. In addition, recent evidence suggests that miRNA may also upregulate translation under certain circumstances [4]. Hundreds of miRNAs have been identified in various species, including *C. elegans*, humans, and the plant *Arabidopsis thaliana*. It is believed that mammalian miRNAs have the potential to regulate at least 20–30% of all human genes [5]. Each miRNA can target up to 200 transcripts directly or indirectly, and multiple miRNAs can target a given gene [6]. Therefore the potential regulatory circuitry afforded by miRNA is extremely complex. The expression of several miRNAs has been shown to be developmentally regulated and several studies have demonstrated that miRNA are responsible for determining cell fate. These results indicate that miRNAs play a major role in fundamental cellular processes, including the timing of cellular development, hematopoiesis, fat metabolism, organogenesis, apoptosis, cell proliferation, and differentiation. There is also strong evidence that miRNAs are implicated in the onset and progression of many diseases, including cancer [3].

Comparison between human cancers and their normal counterparts have revealed distinct miRNA expression profiles. Indeed, a number of studies have reported differentially regulated miRNAs in diverse cancer types such as breast cancer [7], lung cancer [8], chronic lymphocytic leukemia [9], colon cancer [10], thyroid carcinomas [11], pancreatic cancer [12], head and neck cancer [5], prostate cancer [13], pituitary adenomas [14], and ovarian cancer [15]–[18]. Collectively, these studies demonstrate that some human miRNAs are consistently deregulated in human cancer, suggesting a role for these genes in tumorigenesis. Specific over-expression or under expression of certain miRNAs has been shown to correlate with particular tumor types [19]. Overexpressed miRNAs could potentially target tumor suppressor genes, while downregulated miRNAs would theoretically regulate oncogenes. For example, the *let-7* miRNAs, which are down regulated in lung cancer, can negatively regulate the oncogenes RAS and HMGA2, providing a mechanism for the upregulation of these oncogenes [8], [20], [21]. Thus, members of the *let-7* family function biologically as tumor suppressors and their loss is predicted to promote transformation and tumor progression. Conversely, the human miRNA cluster *miR-17-92* acts as an oncogene in B-cell lymphoma [22] and lung cancer [23], and can collaborate with *myc* [22].

Unique miRNA expression signatures have been found to be associated with bio-molecular and prognostic characteristics of human lung cancer and chronic lymphocytic leukemia [8], [24], indicating that miRNA signatures could be used to define biological or clinical features of human cancers. The development of new miRNA markers in the near future will represent one of the main goals in molecular medicine as miRNA expression profiles might better classify poorly differentiated tumors as compared with the transcript-based classifiers [19].

There is a limited number of studies in the literature on the roles of miRNAs in ovarian cancer. Zhang et al. reported high frequency genomic alterations involving miRNA genes in 227 human ovarian cancer, breast cancer and melanoma specimens [15]. In another study, 84 tissues (15 normal and 69 malignant) and 5 cell lines were analyzed for alteration in miRNA profile [16]. Finally a study analyzing 10 ovarian tumors and 10 different normal controls was recently reported [17]. In this report, we describe a series of experiments to elucidate the changes in miRNA expression in ovarian cancer and to identify possible targets of relevant candidate miRNAs.

## Results

### miRNA Expression patterns in ovarian tissues

**Figure 1:**
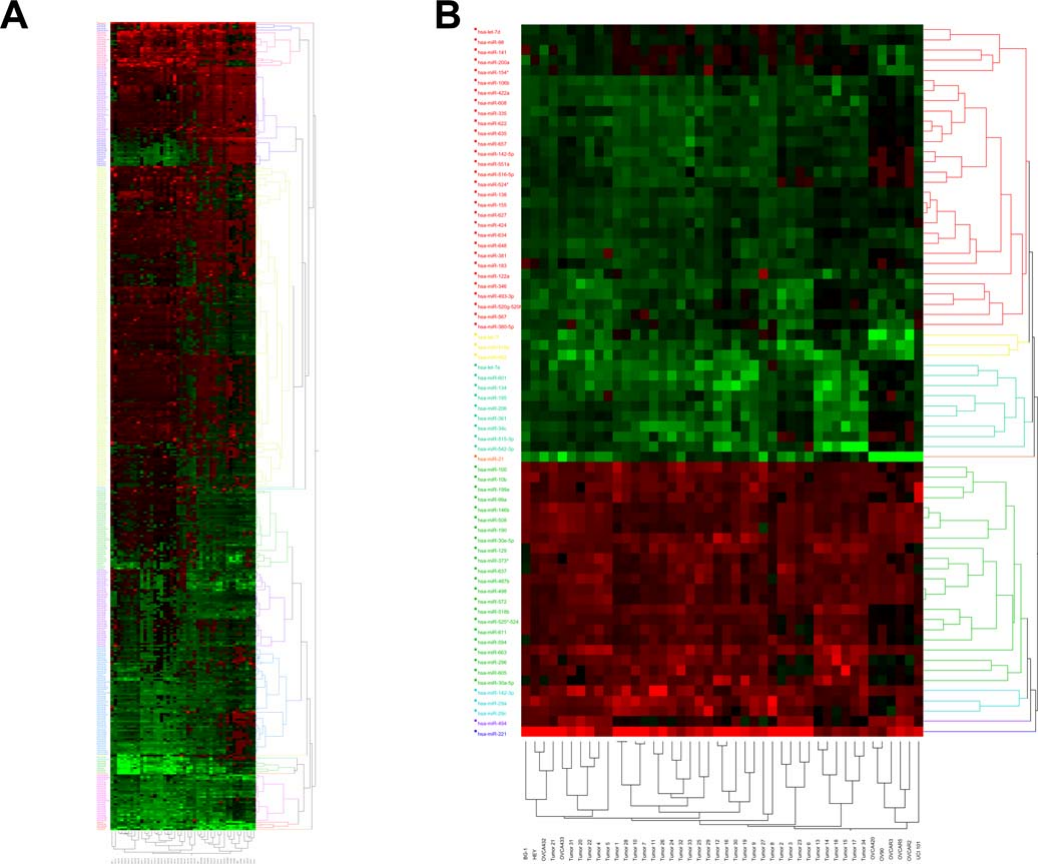
Cluster analysis of miRNA expression. Tree generated by cluster analysis of ovarian cancer tissues and cell lines based on (A) all tested miRNAs in tissues and cell lines, and (B) differentially regulated miRNAs (Fold change >2.0 or <0.5 in greater than 60% of the samples) in tissues and cell lines compared to the normal control HOSE-B cells.

MiRNA expression profiles were determined for 34 ovarian cancer tissues (Table 1) as well as 10 ovarian cancer cell lines. An immortalized human ovarian surface epithelium cell line was used as non-transformed control. The expression profiles were determined using miRCURY™ LNA miRNA Arrays. LNAs are a class of conformationally restricted nucleotide analogs that increase affinity of an oligonucleotide for its complementary RNA or DNA target. The Exiqon miRNA array is currently the most comprehensive probe set available on an array platform and allows for an in depth analysis of miRNA expression. Following RNA hybridization and array analysis, samples were clustered according to their miRNA profile using the hierarchical clustering algorithm of the JMP 6.0.0 software. Figure 1A shows hierarchical clustering analysis of tumor tissues and cell lines based on overall miRNA expression. The profiles shown are relative to ovarian surface epithelial cells (HOSE-B cells). The samples are separated into three main clusters. The middle cluster is comprised entirely of tumors (17 samples), while the left cluster contains 2 cell lines and 7 primary tumors. The right cluster is enriched in cell lines and contains 8 cell lines out of 18 members in the cluster. The hierarchical clustering was then repeated based on a much smaller number of genes and included only 70 highly differentially expressed miRNA in ovarian cancer tissues and cell lines. This clustering analysis gave rise to multiple clusters, but again, the cell lines clustered together in 2 different clusters while the primary tissues were grouped together (Figure 1B), suggesting specific differences in miRNA expression between ovarian cancer tissues and cell lines.

**Table 1:** Patient data.

Sample	General Classification	Age	Grade /Stage
Tumor 1	Borderline carcinoma	57	-
Tumor 2	Serous carcinoma low grade	55	Grade 2, stage 3
Tumor 3	Serous carcinoma low grade	73	Grade 2, stage 3
Tumor 4	Clear cell carcinoma	50	-
Tumor 5	Clear cell carcinoma	72	Grade 3
Tumor 6	Serous carcinoma	53	Stage 4
Tumor 7	Serous carcinoma	57	-
Tumor 8	Serous carcinoma	-	-
Tumor 9	Serous carcinoma	69	-
Tumor 10	Serous carcinoma high grade	76	Grade 3
Tumor 11	Serous carcinoma high grade	74	Grade 3
Tumor 12	Serous carcinoma high grade	64	Grade 3
Tumor 13	Serous carcinoma high grade	66	Grade 3
Tumor 14	Serous carcinoma high grade	69	Grade 3
Tumor 15	Serous carcinoma high grade	48	Grade 3
Tumor 16	Serous carcinoma high grade	46	Grade 3
Tumor 17	Serous carcinoma high grade	86	Grade 3
Tumor 18	Serous carcinoma high grade	52	Grade 3
Tumor 19	Serous carcinoma high grade	64	-
Tumor 20	Serous carcinoma high grade	61	Grade 3, stage 3
Tumor 21	Serous carcinoma high grade	66	Stage 3
Tumor 22	Serous carcinoma high grade	56	Grade 3
Tumor 23	Serous carcinoma high grade	76	Grade 3, stage 3
Tumor 24	Serous carcinoma high grade	64	-
Tumor 25	Serous carcinoma high grade	71	-
Tumor 26	Serous carcinoma high grade	63	Grade 3
Tumor 27	Serous carcinoma high grade	77	-
Tumor 28	Serous carcinoma high grade	68	Stage 3
Tumor 29	Serous carcinoma high grade	80	Grade 3
Tumor 30	Serous carcinoma high grade	64	Grade 3, stage 4
Tumor 31	Serous carcinoma high grade	57	Grade 3, stage 3
Tumor 32	Serous carcinoma high grade	68	-
Tumor 33	Serous carcinoma high grade	64	Grade 4
Tumor 34	Serous carcinoma high grade	51	Grade 4

**Figure 2:**
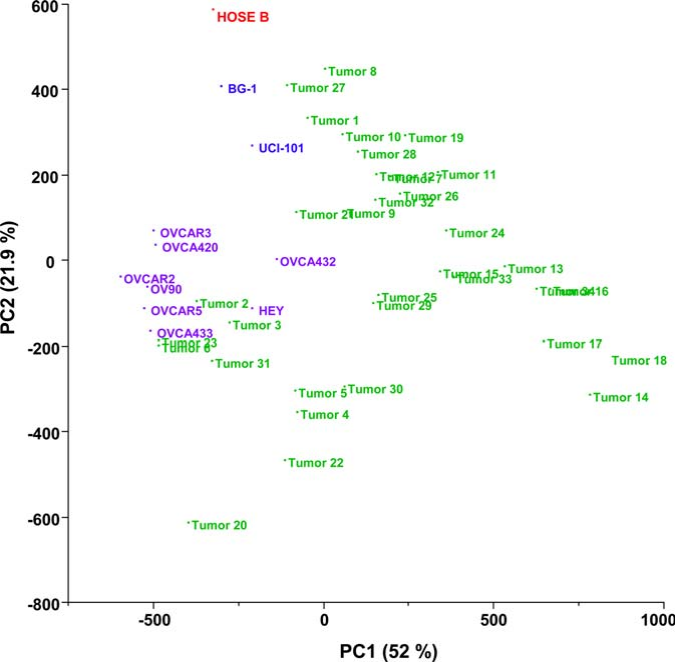
Principal component analysis of ovarian cancer samples (PCA) based on global miRNA expression. Two-dimensional PCA shows that global miRNA expression patterns are different in ovarian cancer cell lines (indicated in blue), ovarian cancer tissues (indicated in green), and the non-tumorigenic HOSE-B cells (in red).

Using principal component analysis (PCA) we found that global miRNA expression could distinguish ovarian cancer tumors from ovarian cancer cell lines (Figure 2). The non-tumorigenic control HOSE-B also exhibited a unique expression pattern, distinguishable from both cell lines and tissues.

### Differentially expressed miRNAs

**Figure 3:**
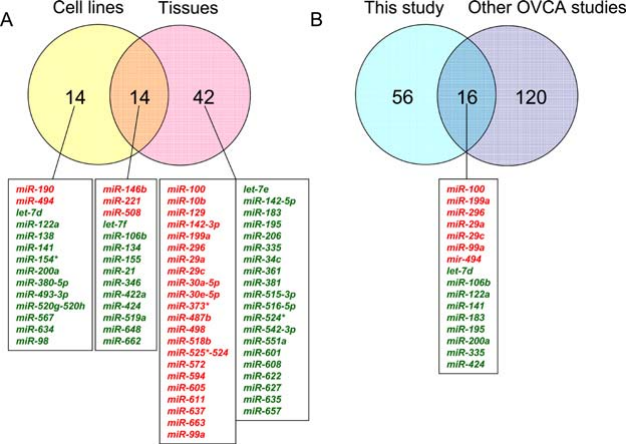
Comparisons of miRNA expression in ovarian tissues. (A) The Venn diagram shows the number miRNAs differentially expressed in ovarian cell lines, in ovarian cancer tissues and in both. For each category, the miRNAs elevated (indicated in red) and downregulated (indicated in green) are indicated below the diagram. (B) The Venn diagram shows the number of differentially expressed miRNAs identified in the current study and the number of miRNAs indentified in 3 previous ovarian cancer studies. The miRNAs in common are indicated below the diagram and color-coded (red: elevated; green: decreased).

We then identified miRNAs that were differentially expressed between non-neoplastic and neoplastic samples (Figure 3A, Table 2). Only miRNAs that were altered at least 2-fold in at least 60% of the samples were considered significant candidates. Using these strict criteria, we identified 25 upregulated and 31 downregulated miRNAs between control and cancer tissues. Similarly, we identified 5 upregulated and 23 downregulated miRNAs in ovarian cancer cell lines compared with non-neoplastic HOSE-B cells. 14 miRNAs were deregulated in both tissues and cell lines and are listed in Figure 3A. The number of miRNAs that showed downregulation in tumor samples (n = 31) as well as in cancer cell lines (n = 23) were higher than the number of upregulated (n = 25 in tumor tissues, n = 5 in cell lines) miRNAs. This is in agreement with previously published miRNA profiling studies, most of which have shown downregulation of miRNAs to be more common than upregulation in cancer [8], [13], [19], [25], [26].

**Table 2:** Differentially expressed miRNAs in ovarian cancer tissues and cell lines.

Gene Name	Fold Change	Predicted Targets (Target Scan/PicTar)
**Tissues: 60% samples, fold > = 2.0 or < = 0.5**		
*mir-221*	9.16	*TCF12, KIT, CDKN1B, RIMS3, AIP1, NAP1L5*
*mir-663*	4.42	*PRRT1, IQSEC2, SCRT1*
*mir-29a*	4.38	*PI15, COL3A1, COL4A4, COL1A1, COL4A5 var 2, COL4A5 var 3*
*mir-142-3p*	4.27	*USP6NL, PRLR, FAM44B, FAM44B, AKT1S1, ARNTL*
*mir-296*	3.85	*LYPLA2, IQSEC2, RNF44*
*mir-30a-5p*	3.80	*CELSR3, FLJ35954, LYRIC*
*mir-30e-5p*	3.72	Not available
*mir-129*	3.60	*TNFSF11* variant 2, *TNFSF11* variant 1, *SOX4*
*mir-518b*	3.43	*MCF2L, WDR1, TSN*
*mir-29c*	3.34	*PI15, COL3A1, COL4A4, CXXC6, C1QTNF6, COL5A3*
*mir-605*	3.20	*VGLL3, PHACTR2, SCAMP1*
*mir-100*	3.16	*THAP2, KBTBD8, C4orf16, SMARCA5, BAZ2A, VLDLR*
*mir-10b*	2.94	*ARSJ, BDNF, TFAP2C, PRKWNK3* variant 2, *PRKWNK3* variant 1, *EPHA8*
mir-373*	2.83	*KPNA4, ATXN7, SYT4*
*mir-199a*	2.78	*ZNF17,DDR1* variant 3, *DDR1* variant 1
*mir-637*	2.71	*RBM9, MNT, DAGLA*
*mir-99a*	2.69	*THAP2, KBTBD8, C4orf16, SMARCA5, BAZ2A, VLDLR*
*mir-487b*	2.66	*NELF, MAP2K4, CDKN2AIP*
*mir-611*	2.66	*RIMBP2, GTDC1, NTRK2*
*mir-594*	2.61	Not available
*mir-572*	2.49	*C14orf101, SAP30BP, ADRBK1*
mir-525*-524	2.39	Not available
*mir-146b*	2.38	Not available
*mir-498*	2.35	*NAP1L3, CXorf1, C9orf5*
*mir-508*	2.26	Not available
mir-524*	0.49	Not available
*let-7f*	0.49	*HMGA2, C14orf28, LIN28B, HMGA2, ARID3B, HIC2*
*mir-424*	0.48	*FGF2, KIF1B, TMEM16C*
*mir-627*	0.47	*USP9X, KIAA1853, CCDC73*
*mir-381*	0.47	*RAB11FIP2, ARID4B, PTCH1, ARID4B, NBEA, IER5*
*mir-183*	0.45	*ITGB1, PFN2, SLAIN1, MAL2, PTPN4, CTDSPL*
*mir-516-5p*	0.44	Not available
*mir-361*	0.43	Not available
*mir-551a*	0.43	*LPHN1, ERBB4, ZFP36*
*mir-142-5p*	0.42	*ZFPM2, FAM18B, FAM91A1, RPS6KA4, AIP1̧ ATP1B1*
*mir-635*	0.41	*NARG1, RUNX2, UBE2D3*
*mir-648*	0.41	*ONECUT2, BTBD7, FUT9*
*mir-515-3p*	0.41	*CLOCK, FAT2, FMNL3*
*mir-542-3p*	0.40	*YPEL5, SR140, RALGPS1*
*mir-155*	0.40	*BACH1, ZNF652, RAB11FIP2, ZNF537, BACH1 variant 2*
*mir-335*	0.39	*P18SRP, HLF, CALU, KIAA0256, CALU, N-PAC*
*mir-206*	0.39	*TNKS2, UST, GJA1, SERP1, TNKS2, POGK*
*mir-657*	0.39	*PRKD3, COPS2, PTPRT*
*mir-195*	0.38	*FGF2, KIF1B, TMEM16C, ARL2, LUZP1, FGF2*
*mir-422a*	0.38	*LOC389834, HECTD2, PRKAR2A*
*mir-21*	0.37	*YOD1, LOC150786, GPR64, PLAG1, RP2, ADNP*
*mir-622*	0.35	*INSIG2, NFYA, ATF2*
*mir-34c*	0.35	*DLL1, VEZATIN, FLOT2*
*mir-106b*	0.35	*EIF5A2, ZNFX1, PKD2, KIAA1404, KIAA1196, ITGB8*
*mir-346*	0.33	*BCL6, ACVR2B, NFIB, KIAA0140, BCL6, ROR1*
*mir-608*	0.31	*DAGLA, EPHA8, SPRY4*
*mir-519a*	0.31	*FLJ31818, TGFBR2, SLAIN1*
*let-7e*	0.31	*HMGA2, C14orf28, LIN28B, HMGA2, ARID3B, HIC2*
*mir-662*	0.30	*C6orf49, NEGR1, MKX*
*mir-134*	0.27	*NIPA1, EML4, PPP1R7, KRAS2 variant b, KRAS2 variant a, ZFPM2*
*mir-601*	0.26	*GOLGA8A, GOLGA8B, GOLGA8E*
**Cell lines: 60% samples, fold > = 2.0 or < = 0.5**		
*mir-221*	7.23	*TCF12, KIT, CDKN1B, RIMS3, AIP1, NAP1L5*
*mir-494*	2.83	*SOCS6, CNR1, ARHGAP5*
*mir-190*	2.59	*OTUD4, NEUROD1, PHF20L1, HSHIN1, STK24, EPC2*
*mir-508*	2.45	Not available
*mir-146b*	2.4	Not available
*mir-424*	0.5	*FGF2, KIF1B, TMEM16C*
*mir-98*	0.5	*HMGA2, C14orf28, LIN28B, HMGA2, ARID3B, HIC2*
*mir-138*	0.5	*FOXC1, RMND5A, SLC35F1, LPHN3, FLJ13910, CLK3*
*mir-380-5p*	0.48	*Not available*
*mir-134*	0.48	*NIPA1, EML4, PPP1R7, KRAS2 variant b, KRAS2 variant a, ZFPM2*
*mir-106b*	0.47	*EIF5A2, ZNFX1, PKD2, KIAA1404, KIAA1196, ITGB8*
*mir-200a*	0.46	*ZEB2, KLF12, ZFR, PPM1E, CUL3, SEC10L1*
*mir-422a*	0.46	*LOC389834, HECTD2, PRKAR2A*
*mir-155*	0.46	*BACH1, ZNF652, RAB11FIP2, ZNF537, BACH1 variant 2*
*mir-122a*	0.44	*GYS1, SLC1A5, 8D6A*
*let-7d*	0.43	*HMGA2, C14orf28, LIN28B, HMGA2, HIC2, KIAA1196*
*mir-141*	0.43	*ZEB2, KLF12, ZFR, PPM1E, IRS2, KIAA1078*
*mir-567*	0.43	*SGMS1, ACLY, GK*
*mir-634*	0.4	*PDIK1L, NRXN3, HIPK1*
*mir-648*	0.39	*ONECUT2, BTBD7, FUT9*
mir-154*	0.38	*ZNF281, PRPF3, C10orf86*
*mir-346*	0.37	*BCL6, ACVR2B, NFIB, KIAA0140, BCL6, ROR1*
*mir-493-3p*	0.36	Not available
*mir-520g-520h*	0.36	*TRIM13, LOC153222, KIAA1826*
*mir-662*	0.3	*C6orf49, NEGR1, MKX*
*mir-519a*	0.26	*FLJ31818, TGFBR2, SLAIN1*
*let-7f*	0.24	*HMGA2, C14orf28, LIN28B, HMGA2, ARID3B, HIC2*
*mir-21*	0.11	*YOD1, LOC150786, GPR64, PLAG1, RP2, ADNP*

Listed, are mRNAs with fold changes >2.0 or <0.5 in at elast 60% of the samples compared to HOSE B cells. *HMGA2, BACH1, BCL6, FAM44B, FGF2, CALU*, and *TNKS2* were predicted by both Pictar and Target Scan for their respective miRNAs.

*miR-221* was the most highly elevated miRNA in both tissues and cell lines (9-fold and 7-fold respectively), while *miR-21* was significantly decreased in both sample types (3-fold and 9-fold, respectively) (Table 2). Among the different *let-7* family members, *let-7e* and *let-7f* showed more than 2-fold deregulation in at least 60% of the tumor samples. The other *let-7* family members (*let-7g*, *let-7d*, *let-7c*, *let-7a-e*, *let-7i*, *let-7a*, *let-7b* were not downregulated as consistently, but each one of them was found decreased 2-fold or more in at least 20% of the tumors. Overall, 94% of the tumors had at least one *let-7* family member downregulated at least 2-fold. Cell lines exhibited downregulation of the *let-7* family members as well. *let-7f* was downregulated more than 4-fold in cell lines, while *let-7d* and *let-7a-e* were also significantly decreased (more than 2.3-fold and 2-fold, respectively).

We then compared the differentially regulated ovarian miRNAs identified in this study with those reported in three previous studies [15]–[17]. Of a total of 136 miRNAs found deregulated in the previous studies, 16 were also identified in our study (Figure 3B). Of these 16 miRNAs, 9 were downregulated (*let-7d*, *miR-106b*, *miR-122a*, *miR-141*, *miR-183*, *miR-195*, *miR-200a*, *miR-335, mir424*) and 7 were upregulated (*miR-100*, *miR-199a*, *miR-296*, *miR-29a*, *miR-29c*, *miR-99a, mir-494*). Interestingly, we report 56 miRNAs that have previously not been found deregulated in ovarian cancer (Figure 3B).

### Predicted gene targets of miRNA genes

miRNAs can regulate a large number of target genes and several databases based on various algorithms are available for predicting the targets of selected miRNAs. Target Scan 3.0 and PicTar were used to predict gene targets of the deregulated miRNAs identified in this study. Table 2 lists the top 3 predicted targets according to both Target Scan and PicTar for each differentially expressed miRNAs. There was little overlap between the targets identified with the two different databases and only 7 targets (FAM44B, BACH1, BCL6, HMGA2, CALU, FGF2, and TNKS2) for 7 miRNAs (*let-7* family, *miR-98*, *miR-155*, *miR-195*, *miR-346*, *miR-206*, *miR-335*) were shared between these two databases when the top 3 targets were examined.

### Experimental identification of miRNA targets

**Figure 4:**
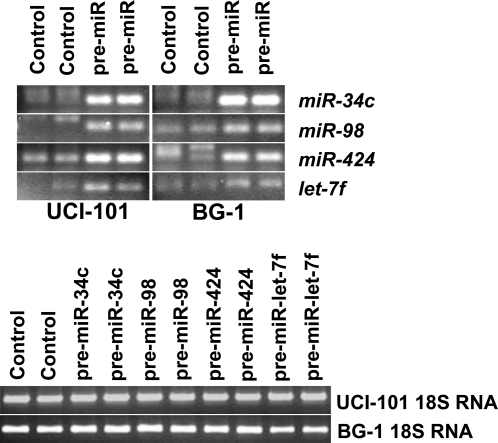
Forced overexpression of selected miRNAs in ovarian cancer cell lines. Pre-*miR-34c*, *Pre-miR-98*, Pre-*miR-424*, Pre-*let-7f* were overexpressed in BG-1 and UCI-101. The products for each of the miRNAs is shown in duplicate for the two cell lines used. Significant overexpression of the miRNAs is confirmed. RT-PCR of 18S RNA is shown for each condition to demonstrate equal loading.

**Figure 5:**
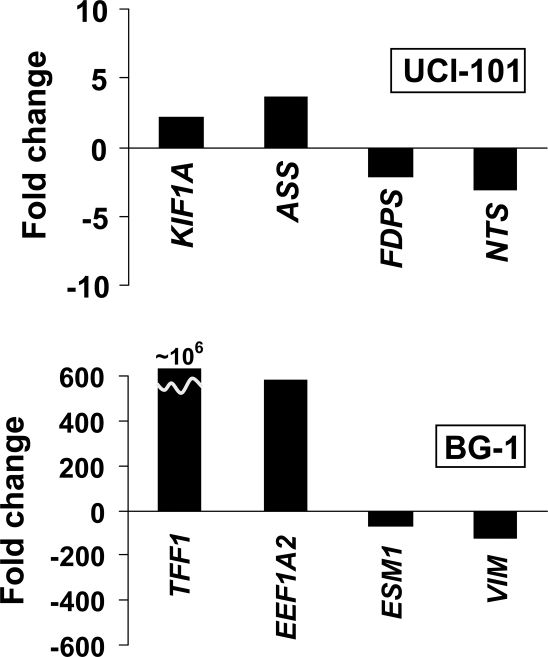
Validation of illumina arrays data for *let-7f*. Transcripts identified by illumina arrays to be altered following let-7f overexpression are validated by RT-PCR. Fold changes for genes *KIF1A, ASS, FDPS, NTS* (in UCI-101 cells), and *TFF1*, *EEF1A2, ESM1, VIM* (in BG-1 cells) are shown and confirm the changes identified by illumina arrays.

To further study the potential targets of key ovarian miRNAs, we overexpressed *miR-34c*, *miR-98*, *miR-424*, and *let-7f* in 2 ovarian cancer cell lines (BG-1 and UCI-101). *miR-424* and *let-7f* were downregulated in both tissues and cell lines (Figure 3A), while *miR-34c* and *miR-98* were found downregulated in ovarian cancer tissues and in cell lines, respectively. Figure 4 shows the successful over-expression of the miRNAs 72 hours following transfection. We then used Illumina microarrays to investigate mRNA level changes following the overexpression of these candidates. Tables 3 and 4 list the transcripts that were most significantly altered following over-expression of the miRNAs in BG-1 and UCI-101 cell lines, respectively. Interestingly, these tables include genes such as *GPX3, MCM7, MSN* that have previously been implicated in ovarian cancer. Among the significantly altered genes (absolute Z-ratio>1.5), there are 10 known cancer genes (*MSN, PIM1, CBL, COL1A1, COX6C, EVI1, EXT1, HLXB9, PTPN11, TCEA1*), 4 tumor suppressor genes (*HIF1A, CAV1, GADD45A, PTTG1IP*), 26 cell cycle genes (*ACAT2, CDK5, FDPS, ID1, LLGL1, MAD2L1, ANLN, ATAD2, C12orf48, CD9, ECT2, GADD45A, GSTO1, HSPD1, IPO7, KNTC2, KRT18, MRPS17, NUDT1, PFN1, PRKCA, SFRP1, SKP2, SNRPA1, VAMP8, WEE1*), and 6 genes involved in chromatin remodeling (*ASF1A, GCN5L2, ATAD2, CBX2, CBX4, NCOA3*). Very little overlap was found between the predicted (Pictar and Target Scan) and experimental targets. Interestingly, the experimental targets varied according to the cell line used, suggesting a significant influence of the molecular background on miRNA target selection. In order to validate the Illumina data, RT-PCR was performed on 8 genes found to be targets of *let-7f* (*KIF1A, ASS, FDPS, NTS* in UCI-101 cells, and *TFF1, EEF1A2, ESM1, VIM*, in BG-1 cells) (Figure 5). We found that, while the absolute values were different, the trends were the same. *KIF1A* and *ASS* were found elevated in UCI-101, while *FDPS* and *NTS* were downregulated in these cells. *TFF1* and *EEF1A2* were confirmed to be elevated in BG-1, while *ESM1* and *VIM* were downregulated.

Using these experimental targets as a starting point (Table 3 and Table 4), we used Ingenuity Pathway Analysis (IPA) to reveal potential diseases, molecular functions, physiological systems, and canonical pathways associated with expression of these miRNAs in BG-1 (Table 5) and UCI-101 (Table 6). Not surprisingly, the analysis identified “cancer” as the main disease associated with these patterns of expression in both cell lines. GI disease was also commonly identified. Interestingly, cellular movement, cell cycle, and cardiovascular functions were systems often found to be related to these expression patterns. There was little consistency in the canonical pathways identified through IPA and except for integrin signaling (found following miR34c or miR98 expression in BG-1), none of the canonical pathways was found more than once in Tables 5 and 6. This analysis therefore suggests that while the general pathways affected by *miR-34c*, *miR-98*, *miR-424*, and *let-7f* expression in these two cell lines are related to cancer, the exact molecular pathways targeted are variable and depend on the cell line and on the miRNAs. This again points to the high level of complexity of miRNA target selection and regulation.

**Table 3:** Top 10 up-regulated and down regulated genes after over expression of mir-34c, mir-98, mir-424 and let-7f in BG-1 cell line.

mir-34c		mir-98		mir-424		let-7f	
Symbol	Z-ratio	Symbol	Z-ratio	Symbol	Z-ratio	Symbol	Z-ratio
*GFPT1*	3.59	*HIST1H4C*	6.34	*AGRN*	4.92	*TFF1*	8.19
*ALOX5AP*	3.41	*CCDC58*	4.44	*BAT2*	4.34	*EEF1A2*	7.89
*NT5C3*	3.36	*ABCA1*	4.28	*GNG12*	4.27	*S100P*	7.61
*SUMF2*	3.33	*RPS7*	4.11	*C20orf149*	4.27	*BMP7*	6.35
*SSR1*	3.28	*ALOX5AP*	4.09	*SUMF2*	4.27	*CRABP2*	5.69
*RND3*	3.25	*SLC38A1*	4.04	*SLC31A1*	4.06	*C3orf57*	5.68
*TSNAX*	3.03	*DBT*	3.92	*NOMO1*	4.03	*PRSS8*	5.64
*C14orf129*	2.96	*NRIP3*	3.88	*PABPC3*	3.97	*KRT19*	5.56
*ASF1A*	2.96	*CUEDC1*	3.87	*ANPEP*	3.96	*FBP1*	5.23
*GTF2H3*	2.90	*C10orf116*	3.82	*WASF2*	3.91	*CLDN3*	5.21
*ECHDC2*	-2.57	*ZNF259*	−3.48	*GCN5L2*	−2.95	*MSN*	−5.96
*MCM7*	−2.59	*WDR58*	−3.54	*ZNF265*	−2.96	*G0S2*	−5.99
*ARHGAP17*	−2.71	*MLF2*	−3.59	*CCDC14*	−3.02	*GPX1*	−6.00
*PIM1*	−2.71	*FLJ90652*	−3.60	*EIF2B5*	−3.04	*TM4SF1*	−6.02
*C8orf13*	−2.74	*GPSN2*	−3.81	*GPX3*	−3.09	*GSTP1*	−6.06
*FASN*	−3.03	*LLGL1*	−4.13	*SLC37A4*	−3.30	*FSTL1*	−6.09
*LEPREL2*	−3.43	*ACTG1*	−4.81	*C16orf48*	−3.35	*IL1A*	−6.49
*C8orf55*	−3.46	*MRCL3*	−5.08	*NPEPL1*	−3.40	*PRG1*	−6.67
*GPX3*	−3.53	*FKSG30*	−5.22	*MAP4K2*	−3.42	*ESM1*	−7.51
*NNMT*	−3.54	*ACTB*	−5.91	*KLF9*	−3.49	*VIM*	−7.78

**Table 4:** Top 10 up-regulated and down regulated genes after over expression of mir-34c, mir-98, mir-424 and let-7f in UCI-101 cell line.

mir-34c		mir-98		mir-424		let-7f	
Symbol	Z-ratio	Symbol	Z-ratio	Symbol	Z-ratio	Symbol	Z-ratio
*FLNC*	5.03	*HIST1H4C*	5.85	*HIST1H4C*	5.27	*KIF1A*	7.89
*MT1A*	4.17	*PSAT1*	4.02	*ID1*	3.65	*ASS*	7.41
*AKNA*	4.1	*ATP5E*	3.93	*IRX3*	3.52	*ASNS*	5.47
*ID1*	3.73	*COX7A2*	3.61	*ID3*	3.4	*C6orf48*	4.77
*MT2A*	3.52	*NDUFA1*	3.58	*EBPL*	3.32	*ATF5*	4.36
*EIF4EBP1*	3.44	*SARS*	3.49	*CXXC5*	2.88	*IGFBP5*	4.36
*KRT10*	3.39	*RPS3A*	3.47	*ATP5E*	2.83	*SFRS5*	4.08
*SARS*	3.34	*RPL14*	3.12	*ALDH9A1*	2.8	*PHGDH*	3.99
*SERPINE1*	3.32	*ERH*	3.03	*KRTCAP2*	2.73	*SOX21*	3.88
*SOST*	3.3	*ATP5O*	2.98	*COX7A2*	2.63	*IARS*	3.73
*UBA2*	−2.56	*IPO4*	−2.5	*CMPK*	−2.7	*ACLY*	−3.56
*DDX39*	−2.62	*OAT*	−2.54	*CLASP2*	−2.8	*C8orf52*	−3.61
*E2F3*	−2.68	*GTF3C3*	−2.66	*ITGAV*	−2.87	*HES6*	−3.97
*HIF1A*	−2.74	*SGK*	−2.74	*C13orf23*	−2.9	*C6orf173*	−4.12
*LOC441087*	−2.74	*HES6*	−2.75	*TSNAX*	−2.96	*DHCR7*	−4.32
*EVI5*	−2.78	*XRCC6BP1*	−2.76	*CAND1*	−3.02	*DHCR24*	−4.61
*COPS2*	−2.78	*HMBS*	−2.78	*TMEM119*	−3.2	*FDFT1*	−4.91
*PVRL3*	−2.83	*NUDCD2*	−2.83	*MAD2L1*	−3.23	*ACAT2*	−5.53
*ALPP*	−2.93	*CDK5*	−3.12	*PIGK*	−3.28	*FDPS*	−5.55
*FDFT1*	−2.96	*TGFBR3*	−3.25	*ARHGAP12*	−3.49	*NTS*	−5.62

**Table 5:** Ingenuity analysis of Genes affected by transfection of miRNAs in BG-1.

	miR34c	miR98	miR424	Let7f
Disease	**Cancer**	**Cancer**	**Cancer**	**Cancer**
	Reproductive System	**GI disease**	**GI disease**	**GI disease**
Molecular and Cellular Function	**Cell Cycle**	Protein Synthesis	**Cellular Movement**	Cell Growth and Proliferation
	Lipid Metabolism	Carbohydrate metabolism	Cellular assembly and organization	**Cellular Movement**
Physiological System	Connective tissue development and function	Hair and Skin development and function	Organism survival	Tissue development and function
	Hematological System	Hepatic system development and function	Connective tissue development and function	**Cardiovascular development and function**
Canonical pathways	Nicotinamide Metabolism	Integrin signaling	Tight Junction Signaling	Aryl Hydrocarbon receptor signaling
	Integrin Signaling	Protein Ubiquitination Pathways	Interferon Signaling	IGF Signaling

The top two pathways are indicated for each parameter. Pathways found more than 3 times between tables 5 and 6 are indicated in bold.

**Table 6:** Ingenuity analysis of Genes affected by transfection of miRNAs in UCI-101.

	miR34c	miR98	miR424	Let7f
Disease	Organismal Injury and abnormalities	Renal and Urological Disease	**Cancer**	**Cancer**
	Hepatic System Disease	**Cancer**	**GI disease**	**GI disease**
Molecular and Cellular Function	**Cellular Movement**	Cellular signaling	**Cellular Movement**	Amino acid Metabolism
	**Cell Cycle**	Cellular Function and Maintenance	**Cell Cycle**	Small Molecule Biochemistry
Physiological System	Tissue Morphology	Hair and Skin development and function	**Cardiovascular development and function**	Nervous system development and function
	Organismal Development	**Cardiovascular development and function**	Tumor Morphology	**Cardiovascular development and function**
Canonical pathways	Aminoacyl t-RNA Biosynthesis	Oxidative Phosphorylation	Oxidative Phosphorylation	Propanoate Metabolism
	Propanoate Metabolism	Glycine	Ubiquinone Synthesis	Aminoacyl t-RNA Biosynthesis

The top two pathways are indicated for each parameter. Pathways found more than 3 times between tables 5 and 6 are indicated in bold.

## Discussion

Previous work has shown that specific miRNA expression signatures in various human cancers can be associated with diagnosis, prognosis, and therapy response [27]. Moreover it has been suggested that specific miRNAs may have crucial roles in the initiation and/or progression of human cancers through their effects on various molecular pathways. A better understanding of miRNA expression in cancer may uncover novel molecular pathways, or novel mechanisms of activation for known pathways. Using the Exiqon miRNA array, the most comprehensive miRNA array available, we have obtained miRNA expression signatures in ovarian cancer and identified several miRNAs differentially expressed in ovarian cancer tissues and cell lines, as well as putative targets for several of these miRNAs.

We have identified a total of 70 miRNAs deregulated in ovarian tissues and 14 of these were also aberrantly expressed in ovarian cancer cell lines (Figure 3A). Overall, there were relatively few miRNAs found in common between this study and previous studies (Figure 3B). There are several possible explanations for this discrepancy. First, the array platform used for the identification of miRNA is different and may yield different patterns. Second, the material we utilized as normal control is different than what was used in previous studies and the choice of normal is known to influence the outcome of gene profiling analysis [28]. We used immortalized ovarian surface epithelial cells while two of the other three miRNA ovarian studies used whole ovaries. We believe that whole ovarian bulk tissue is not the most appropriate normal control as the ovary is composed of several cell types and that the epithelium only represents a small fraction of the ovarian tissue. The use of an immortalized cell strain also has drawbacks, but we believe it to be a better approximation of normal ovarian epithelial tissues than the mixture present in whole ovaries. In any case, the independent validation of expression patterns is crucial, regardless of the source of normal tissue or tumors used in the study.

We identify *let-7f* as highly downregulated in both cell lines and tumors. Expression of various *let-7* miRNAs have been reported to be down regulated in breast cancers [7], lung cancers [29], thyroid cancer [11], prostate cancer [13], and ovarian cancer [15]–[17]. An association between *let-7* downregulation and poor prognosis has been reported in human lung [29] and breast cancer [7]. The finding that the *let-7* family of miRNAs regulates the expression of the RAS oncogene family provides a potential molecular basis for the role of *let-7* miRNAs in human cancer [20]. *let-7* is therefore considered to be a tumor suppressor miRNA and, interestingly, *let-7* genes map to loci deleted in multiple types of cancers, including ovarian cancer [20]. In addition, *let-7f* has been reported to promote angiogenesis by targeting anti-angiogenic genes [30]. The current work as well a previous reports [15]–[17] showing that members of the *let-7* family are altered in ovarian cancer suggest that *let-7* may represent an important player in this disease. It will be important to identify *let-7* downstream targets relevant to ovarian tumorigenesis.

The most consistently and highly upregulated miRNA in both tissues and ovarian cancer cell lines was *miR-221* (Table 2). *miR-221* has been shown to be highly upregulated in pancreatic cancers [12], in glioblastoma [31] and was implicated in thyroid cancer [32]. *miR-221* has been shown to target the oncogene KIT [33] as well as the tumor suppressor p27kip1 [34].

While *miR-21* overexpression has been observed in several cancers, including glioblastoma [31], breast cancer [7], [35], lung cancer [8], pancreatic cancer [36], human malignant cholangiocyte cell lines [37] and colon cancer [38], we found this gene highly downregulated in our samples. As a matter of fact, *miR-21* was the most significant downregulated miRNA in ovarian cancer when our data was averaged across all samples and cell lines. Our finding is counter-intuitive since it has been found that *miR-21* downregulation was associated with increased apoptosis and decreased cell proliferation [35]. *miR-21* was suggested to function as an oncogene modulating tumorigenesis through regulation of genes such as BCL2 [35], PTEN [37] and PDCD4 [39]. We are currently investigating the possible targets of *miR-21* in our cells as well as the possible reasons behind its downregulation in our system. It is possible that the targets of certain miRNAs could be tissue-specific, which would explain differences in the roles of various miRNA in different tissues.

Other miRNAs found differentially expressed in both tissues and cell lines are *miR-146b*, *miR-508*, *miR-106b*, *miR-134*, *miR-155*, *miR-346*, *miR-422a*, *miR-424*, *miR-519a*, *miR-648*, *miR-662*. Several of these miRNAs have been implicated in the other malignancies or in the control of growth and apoptosis. For example, *miR-134* downregulated cell growth, and *miR-155* increased cell growth in lung carcinoma cells, A549 [40]. The contribution of each of these players to ovarian tumorigenesis remains to be determined.

*miR-34c* has recently been shown to be a transcriptional target of p53 [41], [42] and can suppress proliferation and colony formation in soft agar in neoplastic epithelial ovarian cells [42]. Interestingly, we found *miR-34c* deregulated in ovarian cancer tissues and this finding suggests that *miR-34c* may play a role in ovarian tumorigenesis through its role in the p53 pathway.

Because of the tissue specificity of the miRNAs, different sets of miRNAs are likely to be upregulated or downregulated in cancers of different cellular origins, although it has been reported that miRNA signatures of different cancer types especially epithelial would share some individual miRNAs [38]. Of the miRNAs that were reported here to be differentially expressed in ovarian cancer, several have been similarly deregulated in other cancers. Overall, 31 miRNAs identified in the current study have also been reported de-regulated in other cancer (data not shown). Moreover, 16 miRNAs identified here have previously been reported to be altered in ovarian cancer (Figure 3b). More interestingly, we identify 56 miRNAs that have not previously been found differentially expressed in ovarian cancer. These miRNA may play a role in ovarian tumorigenesis and are currently being investigated.

In order to identify potential targets of the differentially expressed miRNAs in ovarian cancer we searched two major databases, PicTar and Target Scan. We found that there was very little overlap between the predicted targets of each algorithm. Indeed, only 7 (FAM44B, BACH1, BCL6, HMGA2, CALU, FGF2, and TNKS2) predicted targets were shared between these two databases. This result illustrates the well-documented difficulties of predicting targets for miRNA [43]. Moreover, it is possible that targets may depend on the cellular environment, (relative ratios of different potential targets may affect the ones most likely to be downregulated) adding another layer of complexity in miRNA target selection. In order to experimentally investigate the changes in mRNA levels, we over-expressed 4 different miRNA candidates (*miR-98*, *miR-424*, *miR-34c* and *let-7f*) in 2 cell lines and assessed transcript levels using an Illumina oligonucleotide array. Clearly, this approach can only identify miRNA targets that have altered mRNA levels (as opposed to translation inhibition targets), but it has previously been shown that miRNAs can downregulate the levels of a large number of transcripts [44]. While some of the transcripts altered may not represent direct targets of the corresponding miRNA, further analysis of individual candidate mRNAs can provide evidence as to whether they represent direct or secondary targets. Ingenuity Pathway Analysis of the targets revealed a number of pathways and system potentially regulated by the overexpressed miRNAs (Table 5 and Table 6). Interestingly, the predicted pathways were not consistent between the 2 cell lines and again suggested that miRNA target selection and therefore, miRNA function is highly tissue dependent.

When the predicted targets (obtained with PicTar and Target Scan) were compared with the experimental targets obtained by Illumina microarray, we observed no overlap. This could be due to a number of reasons. First, the algorithms for the identification of miRNA targets may preferentially identify targets that result in translational repression, which obviously would not be identified by our microarray experiment. Moreover, as discussed previously, it is likely that the miRNA targets are tissue-specific. In agreement with this possibility is the fact that the targets we identified experimentally were completely different in the two cell lines we used. Interestingly, most of the highly up regulated and down regulated (top 10) genes have already been reported to have roles in cancer or other cellular activities related to cancer (Table 3 and 4), such as GSTP1 [45], [46], HIF-1 [47], [48], and SGK [49].

In this study we have identified miRNAs differentially expressed in ovarian cancer. As a first step in determining their roles in ovarian tumorigenesis, we have started to identify targets of these miRNAs. We show that the predicted targets are different from the experimental and that experimental targets vary according to the cellular background in which miRNAs are expressed. It will therefore be important to methodically study each miRNAs in several models in order to clarify their roles in ovarian cancer. In addition, a combination of overexpression/targeting events by miRNA along with protein array technology will be crucial in identifying the miRNA:mRNA players of ovarian cancer. The picture that is likely to emerge is a highly complex set of interactions between miRNAs and target mRNAs. A better understanding of these pathways will bring us yet closer to an understanding of the molecular mechanism underlying this disease and hopefully to novel approaches for detection and therapy.

## Materials and Methods

### Cell Lines and Tissue Samples

Ovarian cancer lines BG-1, UCI-101, HEY, OVCA420, OVCA432, OVCA433, OVCAR2, OVCAR3 and OVCAR5 cells were cultured in McCoy's 5A growth medium (Invitrogen) supplemented with 10% fetal bovine serum (FBS) and antibiotics (100 units/ml penicillin and 100 µg/ml streptomycin). Ovarian cancer cell line OV90 was cultivated in MCBD 105 medium (Sigma)+Medium 199 (Invitrogen) supplemented with 15% FBS and antibiotics. HOSE-B, an ovarian surface epithelial cell line immortalized with E6 and E7 [50], was cultivated in RPMI1640 supplemented with 10% FBS, antibiotics and 5 ng/ml EGF. Eighteen frozen primary ovarian cancers were obtained through the Collaborative Human Tissue Network Gynecologic Oncology Group (Children's Hospital, Columbus, OH). In addition, nine frozen primary ovarian cancer samples and seven RNA samples (from primary ovarian cancer tissues) were obtained from the Department of Pathology of The Johns Hopkins Medical Institutions (Baltimore, MD). Histological classification of all the tissues is presented in Table 1 and all samples were found to contain greater that 80% cancer cells.

### RNA extraction & quantification

Total RNA was obtained using Trizol (Invitrogen, Carlsbad, CA) according to the manufacturer's instructions. RNA was quantified and assessed using the RNA 6000 Nano Kit and 2100 Bioanalyzer (Agilent Technologies UK Ltd, West Lothian, UK).

### miRNA microarray hybridization

miRCURY™ LNA miRNA Arrays (Exiqon), consisting of control probes, mismatch probes and 1458 capture probes, perfectly matched probes for all miRNA in all organisms as annotated in miRBase Release 8.1, July 2006 (human miRBase 8.2 is also covered) were used in this study. The capture probes cover 92.3% of miRNAs annotated in miRBAse 9.0. The control probes includes 10 spike-in control probes to assure optimal labeling and hybridization, eight negative capture probes and twelve capture probes that hybridize to small nuclear RNAs. RNA labeling and hybridization was completed according to the manufacturer's instructions. Briefly, RNA from each sample was labeled with Cy3 using miRCURY™ LNA miRNA Array labeling kit. After labeling, the samples were loaded onto the microarray slide and incubated 16–18 hrs at 60°C. After hybridization the slides were washed, dried by centrifugation, and scanned using the Agilent Microarray Scanner (model G2565B).

### miRNA microarray analysis

Average values of the replicate spots of each miRNA on the microarray were normalized using global normalization. The correction factor was calculated by dividing the sum of intensities of each sample by the average of all the samples. The normalized values were calculated by multiplying average intensities of each miRNA with the correction factor. The expression levels for each tumor sample were then computed relative to the expression observed in the non-tumorigenic HOSE-B cells. The JMP 6.0.0 software (SAS Institute Inc, Cary, NC) was used for hierarchical clustering, generation of heat maps, and principal component analysis (PCA). Agglomerative hierarchical clustering was applied using the complete linkage method to investigate whether there was evidence for natural groupings of tumor samples based on correlations between gene-expression profiles. The miRNA array data is MIAME compliant has been submitted to the NCBI Gene Expression Omnibus (GEO) database (Accession: GSE10150).

### miRNA over-expression in cancer cell lines

Cell lines were tranfected with Pre-miR™ miRNA precursor (Ambion) and control siRNA (Dharmacon) in 24 well plates using siPORT Neo-FX™ (Ambion) according to the manufacturer's protocol. Three days post-transfection, RNA was prepared using RNeasy kit (Qiagen) and changes in RNA levels analyzed using illumina microarrays (below). The changes in miRNA levels were monitored using N-code cDNA synthesis and qPCR.

### Illumina Microarray analysis and validation

Biotinylated cRNA was prepared using the Illumina RNA Amplification Kit (Ambion, Inc., Austin, TX) according to the manufacturer's directions starting with approximately 500 ng total RNA. Samples were purified using the RNeasy kit (Qiagen, Valencia, CA). Hybridization to the Sentrix HumanRef-8 Expression BeadChip (Illumina, Inc., San Diego, CA), washing and scanning were performed according to the Illumina BeadStation 500× manual (revision C). Array data processing and analysis was performed using Illumina Bead Studio software. The illumina microarray data is MIAME compliant has been submitted to the NCBI Gene Expression Omnibus (GEO) database.

Validation of the expression patterns of the following genes KIF1A, ASS, FDPS, NTS (in UCI-101), and TFF1, EEF1A2, ESM1 VIM (in BG-1) was performed using RT-PCR as previously described [51]. Primer sequences are available from the authors. Gnes diffentially expressed following miRNA expression were analyzed using Ingenuity Pathway Analysis (Redwood City, California).
